# 3-D neurohistology of transparent tongue in health and injury with optical clearing

**DOI:** 10.3389/fnana.2013.00036

**Published:** 2013-10-22

**Authors:** Tzu-En Hua, Tsung-Lin Yang, Wen-Chan Yang, Ko-Jiunn Liu, Shiue-Cheng Tang

**Affiliations:** ^1^Connectomics Research Center, National Tsing Hua UniversityHsinchu, Taiwan; ^2^Department of Otolaryngology, National Taiwan University Hospital and National Taiwan University College of MedicineTaipei, Taiwan; ^3^Research Center for Developmental Biology and Regenerative Medicine, National Taiwan UniversityTaipei, Taiwan; ^4^National Institute of Cancer Research, National Health Research InstitutesTainan, Taiwan; ^5^School of Medical Laboratory Science and Biotechnology, Taipei Medical UniversityTaipei, Taiwan; ^6^Department of Medical Science, National Tsing Hua UniversityHsinchu, Taiwan

**Keywords:** neural network, neuromuscular junction, neurohistology, optical clearing, papilla, skeletal muscle, tongue innervation, tongue lesion

## Abstract

Tongue receives extensive innervation to perform taste, sensory, and motor functions. Details of the tongue neuroanatomy and its plasticity in response to injury offer insights to investigate tongue neurophysiology and pathophysiology. However, due to the dispersed nature of the neural network, standard histology cannot provide a global view of the innervation. We prepared transparent mouse tongue by optical clearing to reveal the spatial features of the tongue innervation and its remodeling in injury. Immunostaining of neuronal markers, including PGP9.5 (pan-neuronal marker), calcitonin gene-related peptide (sensory nerves), tyrosine hydroxylase (sympathetic nerves), and vesicular acetylcholine transporter (cholinergic parasympathetic nerves and neuromuscular junctions), was combined with vessel painting and nuclear staining to label the tissue network and architecture. The tongue specimens were immersed in the optical-clearing solution to facilitate photon penetration for 3-dimensiontal (3-D) confocal microscopy. Taking advantage of the transparent tissue, we simultaneously revealed the tongue microstructure and innervation with subcellular-level resolution. 3-D projection of the papillary neurovascular complex and taste bud innervation was used to demonstrate the spatial features of tongue mucosa and the panoramic imaging approach. In the tongue injury induced by 4-nitroquinoline 1-oxide administration in the drinking water, we observed neural tissue remodeling in response to the changes of mucosal and muscular structures. Neural networks and the neuromuscular junctions were both found rearranged at the peri-lesional region, suggesting the nerve-lesion interactions in response to injury. Overall, this new tongue histological approach provides a useful tool for 3-D imaging of neural tissues to better characterize their roles with the mucosal and muscular components in health and disease.

## Introduction

Tongue receives extensive innervation from the cranial nerves to perform taste, sensory, and motor functions (Mu and Sanders, 2010). Tongue is also regulated by the autonomic nervous system, consisting of the sympathetic and parasympathetic nerves to stimulate salivation (Aps and Martens, 2005). In the oral cavity, the tongue epithelium and nerves are in constant contact with high concentrations of growth and neurotrophic factors in the saliva, which stimulate mucosal regeneration as well as potentiate the neural activity in response to gustatory cues (Zelles et al., 1995; Nosrat, 1998; Nosrat et al., 2012).

Due to the rich innervation of oral tissues, patients with tongue injury, such as the oral cancer patients, suffer from pain which is often more severe than that caused by other cancers (Dios and Leston, 2010; Viet and Schmidt, 2012). This is likely due to the stimulation of the nerve endings and/or compression and invasion of sensory nerves. Also, the neural regulation of tongue movements is crucial for speech and swallowing, as the lack of neuromuscular control in tongue dysfunction could lead to dysarthria and dysphagia (Hiiemae and Palmer, 2003; Miller, 2008). Despite the noticeable symptoms involved with the nervous system, high-resolution microscopy of tongue innervation and its remodeling in response to pathophysiological cues has been difficult. This is primarily due to the dispersed neural network that cannot be portrayed easily by the standard microtome-based 2-dimensional (2-D) histology (Berlanga et al., 2011). The artifacts caused by microtome slicing and the challenge of aligning series of microtome slices limit our ability to examine tongue innervation in a 3-dimensional (3-D) space continuum.

The standard histological and immunohistochemical analyses of tongue specimens are performed on the microtome sections (El-Rouby, 2011; Krishnan et al., 2012), approximately 5 μm in thickness, to avoid light scattering for optical microscopy. However, this dimension of thickness is one order of magnitude smaller than the size of a papilla and 2~3 orders of magnitude smaller than a typical tongue lesion or biopsy. Although the sectioned tongue allows sufficient light transmission, the 2-D tissue slice offers only a limited view of the innervation.

To overcome the imaging hurdle, we previously developed a penetrative imaging method, based on preparation of transparent tissues (or “optical clearing:” use of the immersion solution to reduce scattering as light travels in the specimen) (Tuchin et al., 2008; Tseng et al., 2009; Fu and Tang, 2010; Fu et al., 2010), for 3-D imaging of the mouse and human gastrointestinal tissue networks (Fu et al., 2009, 2013; Chiu et al., 2012; Liu et al., 2012, 2013; Tang et al., 2013), including the enteric nervous system (Liu et al., 2011; Smith, 2011). Here, in tongue neurohistology, we employed the same 3-D imaging approach to provide a global view of the tongue microstructure and innervation.

In addition to the normal tongue, in this research we incorporated a disease model of tongue dysplasia induced by 4-nitroquinoline 1-oxide administration in the drinking water of mice to study the neural tissue remodeling in response to tongue injury. The carcinogen 4-nitroquinoline 1-oxide causes oral mucosal damage by exerting intracellular oxidative stress and disturbing the DNA structure through its metabolic product (Vered et al., 2005; Kanojia and Vaidya, 2006). The pathogenic mechanisms are similar to those induced by carcinogens in tobacco, a primary risk factor for oral cancer. In the oral lesion progression, white or red plaques appear in the oral cavity 8 weeks after exposure to 4-nitroquinoline 1-oxide. Mild or moderate dysplasia in the tongue appears around 12–18 weeks in almost all mice. Afterward, severe dysplasia (our focus in this research) with large tongue lesions and in some mice carcinoma *in situ* and squamous cell carcinoma can be observed. In the process, whether or not the neural tissue plays a role in cancer development is unclear. Here, for the first time we used 3-D neurohistology to identify and illustrate the remodeling of mouse tongue innervation in association with the 4-nitroquinoline 1-oxide-induced dysplasia. The development of our panoramic tongue imaging approach and the morphological and pathophysiological implications of the neural tissue network in health and disease are presented and discussed.

## Materials and methods

### Animals

Normal tongues were harvested from BALB/c mice (BioLASCO, Taipei, Taiwan), age 16–20 weeks, to perform analysis of optical clearing and study the clearing effect on deep-tissue microscopy. C57BL/6 mice, age 8 weeks, were treated with 4-nitroquinoline 1-oxide (Sigma-Aldrich, Saint Louis, MO, USA)—a carcinogen used to develop oral/tongue dysplasia and cancer (Kanojia and Vaidya, 2006)—in drinking water at a concentration of 200 μg/ml for 16 weeks to develop oral lesions and were sacrificed 12 weeks afterward. We focused on tongue dysplasia to investigate the nerve-lesion association. All animal procedures were approved by the Institutional Animal Care and Use Committee of the National Health Research Institutes. Eight normal and eight diseased mice were used to generate representative images. Note: due to the potential variations in tongue pigmentation, we chose to demonstrate that the tongues from the white BALB/c and black C57BL/6 mice can both be optically cleared for deep-tissue microscopy.

### Preparation of tongue specimen

Tongue blood vessels were labeled by cardiac perfusion of the lectin-Alexa Fluor 488 conjugate (30 μ g/g of body weight, Invitrogen, Carlsbad, CA, USA) (vessel painting) followed by 4% paraformaldehyde perfusion fixation (Fu et al., 2013). Afterward, the perfused tongue was harvested and cryosectioned (200 μm in thickness). The tongue cross-sections were post-fixed in 4% paraformaldehyde solution for one hour at 25°C and then immersed in 2% Triton X-100 solution for 2 h at 25°C for permeabilization.

**Figure 1:**
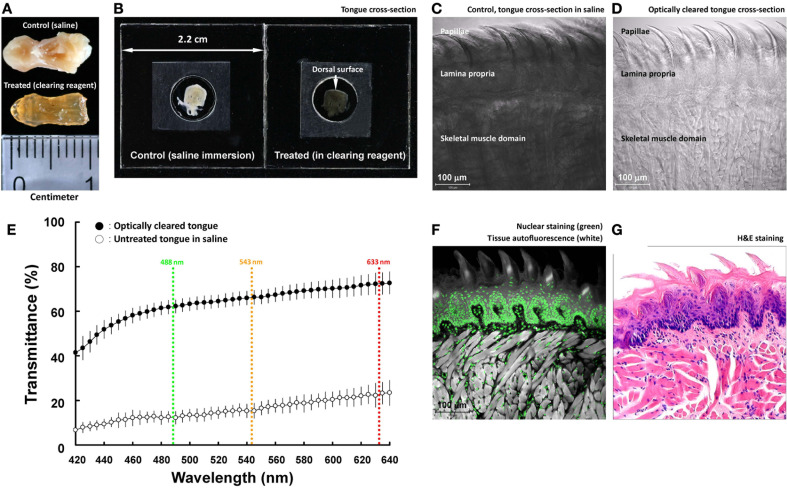
**Optical clearing increases light transmission of mouse tongue specimens. (A,B)** Change of tongue optical property after the clearing process. The entire tongue (panel **A**) and cross-section (panel **B**; thickness: 200 μm) were immersed in saline and optical-clearing solution prior to taking the images. Solutions were removed in panel **(A)** to avoid light reflection. In panel **(B)**, the specimen was held by two coverslips and a spacer filled with immersion solution. **(C,D**) Transmitted light micrographs of the tongue specimens in saline and optical-clearing solution. **(E)** Increase in light transmission across the optically cleared tongue cross-section over a spectrum of wavelengths. Colored lines indicate the wavelengths of laser lines used in confocal microscopy. Results are presented as mean ± standard deviation (*n* = 8). **(F,G)** Confocal micrograph of the optically cleared tongue and the standard H&E image. The confocal micrograph consists of fluorescence signals derived from nuclear staining (propidium iodine) and tissues' autofluorescence (excited by the 488-nm laser). Both the confocal and H&E images show the tongue mucosal and muscular structures.

Four different primary antibodies were used to immunolabel the neural tissues following the protocol outlined below. The antibodies used were a rabbit anti-PGP9.5 (Epitomics, 2932–1, Burlingame, CA, USA), rabbit anti-CGRP (Sigma-Aldrich, C8198), rabbit anti-tyrosine hydroxylase (Millipore, AB152, Billerica, MA, USA), and rabbit anti-VAChT (Synaptic Systems, 139103, Gottingen, Germany) antibodies (Zhang et al., 2010). The tissue was first rinsed in PBS and then incubated with the blocking buffer (2% Triton X-100, 10% normal goat serum, and 0.02% sodium azide in PBS). The primary antibody was then diluted (1:50) in the dilution buffer (0.25% Triton X-100, 1% normal goat serum, and 0.02% sodium azide in PBS) and incubated with the tissue for one day at 15°C. An Alexa Fluor 647 conjugated goat anti-rabbit secondary antibody (1:200, Invitrogen) was then used to reveal the immunopositive structure. Afterward, propidium iodide staining (50 μg/ml) was performed at room temperature for one hour to label the nuclei. Immunohistochemistry and nuclear staining were performed on free floating sections. The labeled specimens were immersed in the optical-clearing solution FocusClearTM (CelExplorer, Hsinchu, Taiwan) at 25°C on an orbital shaker for 2 h and then sealed between a pair of coverslips with a spacer filled with the same solution overnight (Figure 1B, right) before confocal microscopy (Carl Zeiss, 2009). Quantitation of the optical-clearing effect was performed using a microplate reader (SpectraMax M2e; Molecular Devices, Sunnyvale, CA, USA) to measure the light transmission.

### Confocal microscopy

**Figure 2:**
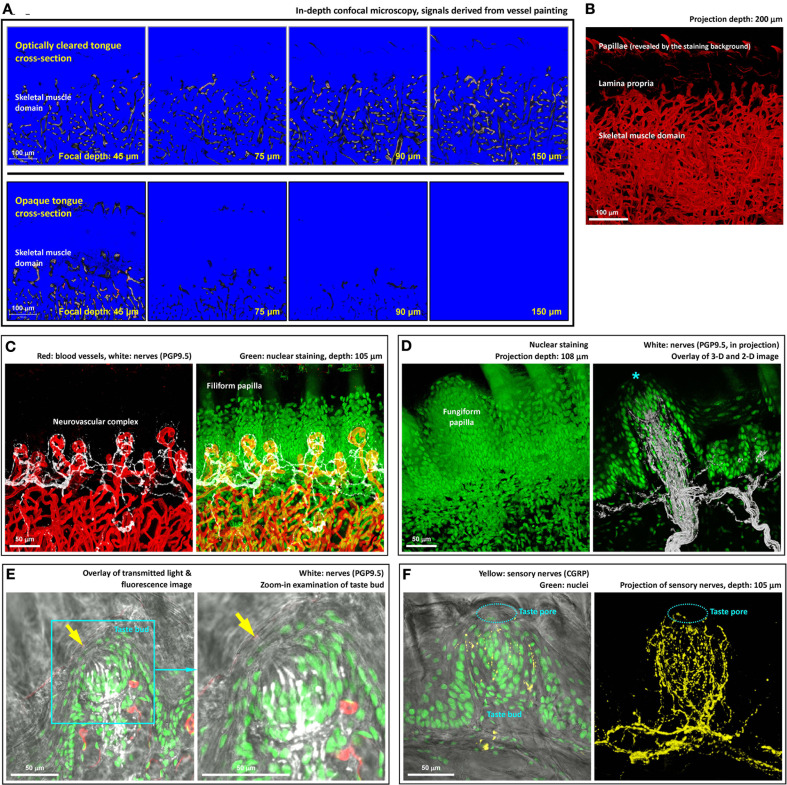
**Deep-tissue microscopy of the tongue microstructure, vasculature, and innervation with high definition. (A)** Extended imaging depth in tongue microscopy with optical clearing. Upper panels show the signals of blood vessels in the optically cleared tongue cross-section, while in the opaque specimen (lower panels) the signals drastically declined as the focal plane progressed into the tissue. The blue and red in the images are the range indicators of signal intensity, showing the locations with no signals and saturated signals, respectively. **(B)** In-depth projection of the vascular signals derived from the optically cleared tongue. 360° presentation of the image stack is shown in Video S3. **(C)** Neurovascular complex in the core of filiform papilla. Video S4 shows a 360° projection of the image stack. **(D)** Microstructure and innervation of fungiform papilla. Left: in-depth projection of a fungiform papilla in between the filiform papillae. Right: projection of the fungiform papilla and its innervation. Asterisk indicates the taste bud at the top of the papilla. A 2-D micrograph is placed at the background to indicate the location of the epithelium. A fly-through presentation of the image stack is shown in Video S5. **(E,F)** Zoom-in examination of taste buds in transparent tongue epithelium. Arrows in panel **(E)** and squashed circles in panel **(F)** indicate taste pores, which connected the exterior and interior domains of the epithelium. Pan-neuronal marker PGP9.5 and sensory nerve marker CGRP were used to reveal the taste bud innervation. Green: nuclei. Red: capillaries. White: PGP9.5-labeled nerves. Yellow: CGRP-labeled sensory nerves. A 360° projection of the sensory nerve network is shown in Video S6

**Figure 3:**
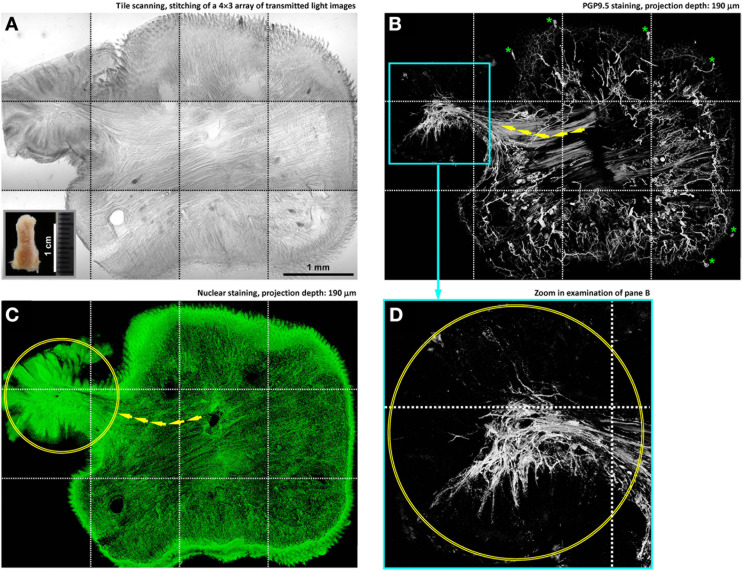
**In-depth microscopy with tile scanning reveals nerves remodeling in response to tongue injury. (A–C)** Stitching of transmitted light and confocal micrographs across the tongue section. Panel **(A)** shows a protrusive lesion at the left side of the section (inset: gross view of the diseased tongue). Pan-neuronal marker PGP9.5 (panel **B**) was used to reveal the tongue innervation. Overlay of panels **(A,B)** is shown in Figure S1. Arrows in panels **(B,C)** indicate the remodeled skeletal muscle fibers—revealed by the staining background and confirmed in the transmitted light micrograph—entering the lesion domain (circles in panels **C,D**). Asterisks in panel **(B)** indicate the landmark fungiform papillae and their innervation (positive control of PGP9.5 staining). **(D)** Zoom-in examination of the condensed PGP9.5^+^ nerve fibers in the lesion domain (the cyan box in panel **B**).

**Figure 4:**
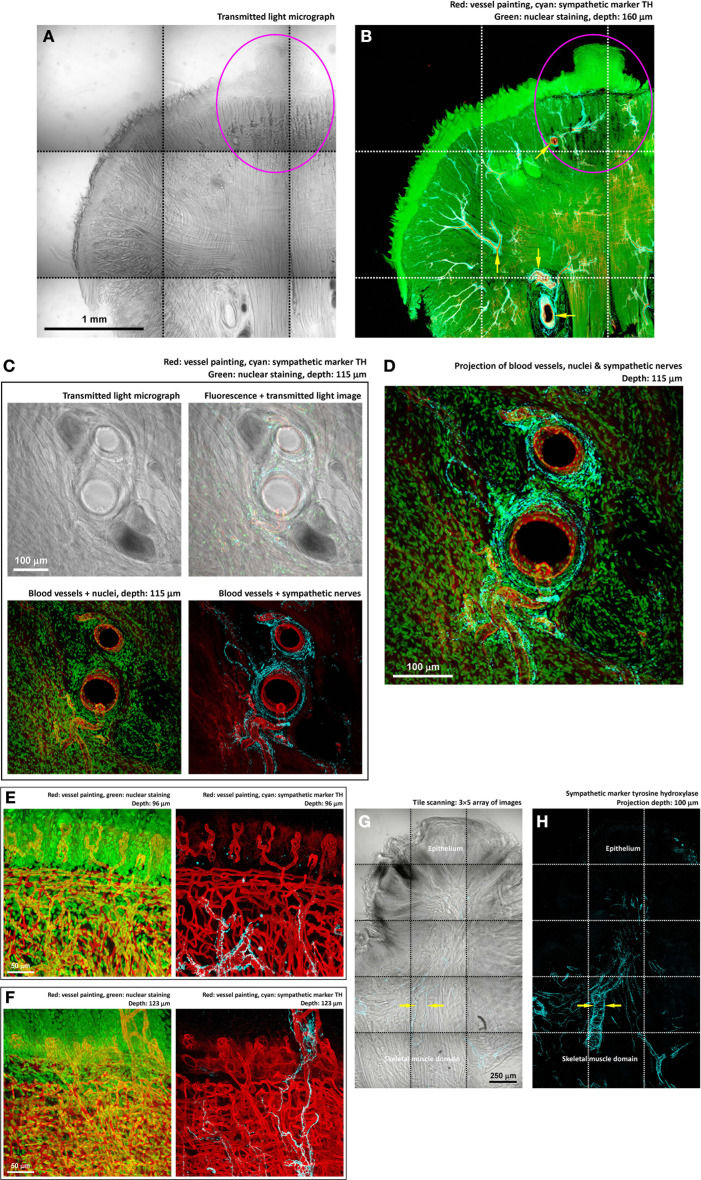
**Perivascular sympathetic innervation of mouse tongue in injury. (A,B)** Gross view of the 4-nitroquinoline 1-oxide-treated tongue. Squashed circles indicate an early-stage lesion. In the gross view, perivascular sympathetic innervation of tongue arterioles was prominent in both the diseased and normal domains (arrows in panel **B**). Cyan: sympathetic marker TH. **(C,D)** Zoom-in examination of sympathetic nerves encircling the tongue arterioles. The morphology indicates the sympathetic control of local blood flow. **(E,F)** Zoom-in examination of perivascular sympathetic innervation of capillaries in tongue mucosa (two examples). Particularly, in panel **(F)**, the in-depth projection shows the sympathetic nerves ascending from the skeletal muscle domain to innervate the fungiform papilla in the mucosa. **(G,H)** TH-labeled sympathetic nerves encircling the arteriole underneath the diseased epithelium. Arrows indicate the perivascular sympathetic innervation.

**Figure 5:**
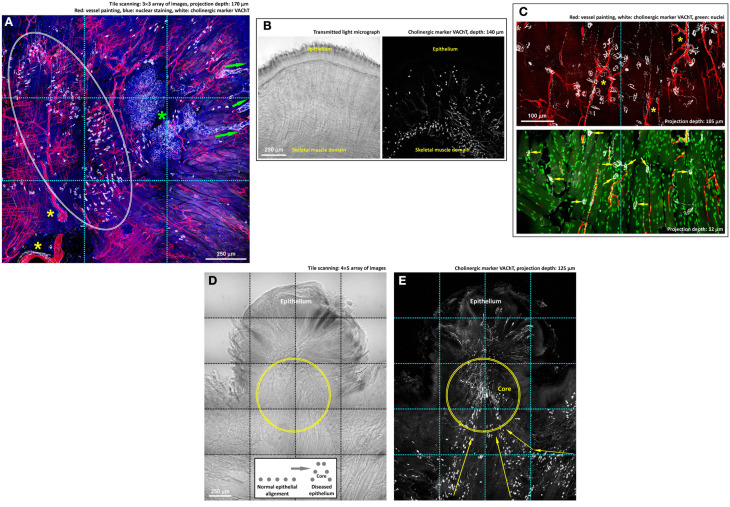
**Cholinergic innervation of mouse tongue and its remodeling in injury.** **(A,B)** Gross view of the VAChT-labeled cholinergic nerves in the tongue. Panel **(A)** reveals three features: (1) the association of the VAChT^+^ parasympathetic varicosities and axons with the von Ebner's gland (green asterisk) and its secretory ducts (arrows indicate the ducts extending toward the mucosal surface), (2) the association of the parasympathetic varicosities with large blood vessels (yellow asterisks), and (3) the aggregation of VAChT^+^ neuromuscular junctions in the skeletal muscle domain (squashed circle). The same aggregation and alignment of tongue neuromuscular junctions can be seen in panel **(B)**, in which the transmitted light micrograph shows the muscle fibers. Some VAChT^+^ neuromuscular junctions were found scattered under the submucosa, indicating the extension of the muscle fibers to this area. **(C)** Zoom-in examination of cholinergic nerves in the tongue muscle domain. The paired staining and projection reveal the perivascular parasympathetic nerves with VAChT^+^ varicosities following the capillaries (asterisks, upper panel) and the cholinergic neuromuscular junctions (arrows, lower panel). **(D,E)** Imaging of the VAChT-labeled cholinergic nerves underneath the diseased epithelium. Panel **(D)**: transmitted light micrograph of a tongue lesion; inset: illustration of the protrusive epithelial remodeling. Panel **(E)**: abundant VAChT^+^ parasympathetic nerve fibers and neuromuscular junctions underneath the tongue lesion. Arrows indicate the remodeled alignment of the VAChT^+^ neuromuscular junctions. Geometrically, the protrusive lesion creates a “core” (illustrated in the inset and marked as the circles in the images) with condensed nerve density, similar to the intra-lesional region shown in panels **3B,D**.

Imaging of the tissue structure was performed with a Zeiss LSM 510 Meta confocal microscope (Carl Zeiss, Jena, Germany) equipped with the objectives of 10 × “Fluar” lenses (optical section: 10 μm; Z-axis increment: 5 μm; applied to acquire Figures 3, 4A,B, 5B), 25 × LD “Plan-Apochromat” glycerine immersion lenses (working distance: 570 μm) (optical section: 5 μm; Z-axis increment: 2.5 μm; applied to acquire Figures 1C,D,F, 2A,B, 4C,D,G,H, 5A,D,E), and 40 × LD “C-Apochromat” water immersion lenses (working distance: 620 μm) (optical section: 3 μm; Z-axis increment: 1.5 μm; applied to acquire Figures 2C–F, 4E,F, 5C) under a regular or tile-scan mode (with automatic image stitching). The laser-scanning process was operated under the multi-track scanning mode to sequentially acquire signals, including the transmitted light signals. The Alexa Fluor 647-labeled structures were excited at 633 nm and the fluorescence was collected by the 650–710-nm band-pass filter. The propidium iodide-labeled nuclei were excited at 543 nm and the signals were collected by the 565- to 615-nm band-pass filter. The lectin-Alexa Fluor 488-labeled blood vessels were excited at 488 nm and the fluorescence was collected by the 500–550-nm band-pass filter.

### Image projection and analysis

The LSM 510 software (Carl Zeiss) and the Avizo 6.2 image reconstruction software (VSG, Burlington, MA, USA) were used for projection and 3-D presentation of the confocal images. Projections in Figures 2B–D,F, 3B–D, 4B–F,H, 5A–C,E were derived from the projection module of the LSM 510 software. In Supplemental Videos, image stacks were recorded using the “Movie Maker” function of Avizo with the increase in display time in association with the depth of the optical section. The 360° presentations in Videos 2–4, 6, and 7 were derived from the “Panorama” function of the LSM 510 software. In Video 5, the “Demo Maker” function of Avizo was used to arrange the sequence of the image objects at different time intervals. The Camera Path function was used to adjust the projection angles and zoom-in and zoom-out movements of the 3-D images.

## Results

### Optical clearing facilitates photon penetration in tongue specimen

Unlike the transparent retina, tongue consists of the mucosa and muscles that strongly scatter light. To facilitate photon penetration in the mouse tongue, we immersed the specimen in the optical-clearing solution (refractive index at ~1.46, similar to that of the tongue tissue constituents) to increase light transmission by avoiding scattering. Figures 1A,B are a comparison of the paraformaldehyde-fixed mouse tongue treated with saline and the optical-clearing solution to demonstrate the change of the tissue optical property after the clearing process. The transparent tissue enabled the use of transmitted light microscopy to observe the tongue microstructures without staining (Figures 1C,D).

The optical-clearing effect was quantified by measuring the light transmission across the tongue cross-section (200 μm in thickness). Specifically, Figure 1E shows that when the wavelengths were at 488, 543, and 633 nm (three common laser lines used in confocal microscopy), the clearing process increased the transmittance from 12, 15, and 23 to 62, 66, and 72%, respectively, or by 4.2-fold on average. The result highlights the improved efficiency of photon penetration in the optically cleared tissue.

Because of the improved transparency, when the tongue was excited by the 488-nm laser, the induced autofluorescence, albeit at low intensity, revealed the tissue morphology in confocal imaging (Figure 1F). When paired with nuclear staining, the acquired confocal micrograph was comparable to the image derived from the H&E staining (Figure 1G). The result indicates that the clearing process changed the tongue optical property but not the morphology.

### Optical clearing enables deep-tissue tongue microscopy

Next, we used signals of tongue microvessels to evaluate the influence of optical clearing on deep-tissue microscopy. Figure 2A compares the confocal images derived from the optically cleared and opaque tongue as the focal depth reached 45, 75, 90, and 150 μm under the tissue surface. As can be seen, while the blood vessels in the optically cleared tongue remained visible at 150 μm, a drastic decline of the fluorescence signals in the opaque tongue was encountered as the focal depth reached 75 μm under the surface. In Figure 2B, we took a series of the confocal images over a span of 200 μm for projection to present the tongue vasculature in space. Additional examples of the in-depth visualization and 360° panoramic projection of the tissue architectures are illustrated in Videos S1–3.

### Visualization of the tongue innervation with high definition

We next combined 3-D microscopy with immunohistochemistry to simultaneously visualize the tongue microstructure, vasculature, and innervation. Figure 2C shows the neurovascular complex at the core of the filiform papilla with the neural network labeled by the pan-neuronal marker PGP9.5 (Wakisaka et al., 1996) (a 360° projection was presented in Video S4). The same approach was also used to visualize the fungiform papilla (Figure 2D and Video S5) and taste bud innervation (Figures 2E,F and Video S6). As can be seen, the transparent tongue mucosa allowed an in-depth imaging of the mucosal structures with the sensitivity and resolving power to identify the taste bud and its pore. Transmitted light and confocal microscopy were combined with nuclear and neuronal marker staining (PGP9.5 or sensory nerve marker calcitonin gene-related peptide, CGRP) (Witt and Reutter, 1998) to reveal the taste bud microstructure and innervation with subcellular-level resolution (use of resolving adjacent nuclei as the criterion). The result demonstrates the resolving power of this new tongue histological approach to characterize the spatial features of the neural and vascular networks.

### Remodeling of tongue microstructure and innervation in response to injury

Figure 3 illustrates the imaging strategy to examine the diseased tongue to identify the remodeling of tongue microstructure and innervation in response to injury. In this task, administration of 4-nitroquinoline 1-oxide in the drinking water was used to induce tongue lesion/dysplasia in mice (Kanojia and Vaidya, 2006). Large-area visualization of the diseased tongue was made possible by image stitching, which allowed us to characterize the tongue morphology across the normal and diseased domains.

Specifically, in the diseased domain (Figures 3A–C, left side and Figure S1) a protrusive tongue lesion consisting of the epithelium and muscle fibers was seen in comparison with the normal, organized tongue architecture at the right side of the image. Underneath the protrusion, condensed nerve fibers followed the muscle fibers into the lesion (Figure 3B, left side), changing from the dispersed neural network in the normal tongue mucosa. The increase in the peri-lesional neural tissues suggests an injury-induced nerve outgrowth. Additional examples of the nerve-lesion contacts are presented in Figure S2 and Video S7.

### Components of tongue innervation in response to injury

We next sought to use markers of neurotransmitters to analyze the components of the tongue innervation. First, we applied immunostaining of tyrosine hydroxylase (TH, sympathetic marker) (Raju et al., 2007; Raju and Ibrahim, 2011) to reveal the sympathetic neural network. The hallmark of the tongue sympathetic innervation is the perivascular presence of sympathetic nerve fibers and their encircling of the arteriole in the muscle domain (Figures 4A–D). The sympathetic nerves also follow the blood vessels entering the mucosa and innervate the fungiform papillae (Figures 4E,F).

Importantly, through tile scanning and in-depth projection, Figures 4G,H show the association of sympathetic nerves with the lesion, particularly underneath the remodeled epithelium. At this location, the arteriole is encircled by the TH^+^ nerves, suggesting the sympathetic control of blood flow into the diseased area.

Second, we applied immunostaining of vesicular acetylcholine transporter (VAChT, marker for cholinergic nerves) to reveal the parasympathetic innervation and neuromuscular junctions of the tongue, both of which employ acetylcholine as the neurotransmitter (Arvidsson et al., 1997; Sbarbati et al., 2002; Maeda et al., 2004). Figure 5A shows a gross view the tongue cross-section to illustrate the cholinergic nerves in the mucosal and muscular domains. Specifically, in and close to the mucosal domain the VAChT-labeled parasympathetic varicosities were associated with the von Ebner's gland and the ducts, indicating parasympathetic regulation of the gland secretion (positive control of the VAChT staining). In the muscular domain, we observed the perivascular presence of the VAChT^+^ parasympathetic varicosities, similar to the perivascular TH^+^ sympathetic innervation.

In the muscular domain, both Figures 5A,B show the VAChT-labeled neuromuscular junctions (VAChT^+^ foci) in addition to the parasympathetic innervation (slender VAChT^+^ fibers). The VAChT^+^ foci were primarily found at the border of the skeletal muscle domain with few scattered underneath the epithelium. Figure 5C shows the zoom-in views of the perivascular parasympathetic innervation and the VAChT^+^ nerve terminals (foci) following the orientation of skeletal muscle fibers; the latter have been described as a morphological marker of the motor nerve innervation (Maeda et al., 2004).

Importantly, underneath the protrusive tongue epithelium, the VAChT^+^ neural tissues, including both the parasympathetic nerve fibers and VAChT^+^ motor nerve terminals, were concentrated at the “core” of the lesion (Figures 5D,E). In particular, the presence of the abundant neuromuscular junctions indicates the coordination of the nerves and muscles around the diseased epithelium in tissue remodeling. Additional examples of the tongue cholinergic innervation and its remodeling in response to lesion formation are presented in Figure S3.

## Discussion

Neural tissues are prominent in the tongue but their structures and remodeling in response to injury have been difficult to study. The challenge is partly due to the lack of appropriate imaging tools to characterize the spatial features of the delicate tongue architectures. In this research, we prepared transparent mouse tongue by optical clearing for penetrative microscopy to visualize the tongue neural networks with high definition. Unlike the standard microtome-based 2-D tissue analysis, our imaging approach provides in-depth anatomic information to illustrate the tongue microstructure, vasculature, and innervation in a global and integrated fashion.

Taking advantage of the optical clearing technique (Figure 1), we overcame the depth limitation imposed by light scattering while imaging the tongue specimen (Figure 2A and Videos S1–S3). Importantly, in addition to the nerves, tissue information acquired from the nuclear and vascular staining and transmitted light microscopy was simultaneously used to create a connected view of the neural network with the associated microstructure and vasculature. For example, in Figures 2C, 4, 5A,C, we revealed the tongue neurovascular complex in papillae and the perivascular autonomic innervation. The in-depth and multi-channel displays of the normal and diseased tongues allowed us to verify the fidelity of the image signals by comparing the sources of information, which is crucial for future clinical evaluation of oral biopsies.

In Figures 3–5, imaging of the 4-nitroquinoline 1-oxide-induced tongue lesions was used to demonstrate the feasibility of performing 3-D microscopy of the diseased tongue. In oral cancer, neurotrophic factors (such as the nerve growth factor) have previously been suggested to regulate cancer/lesion progression, pain, and cachexia (Kolokythas et al., 2010; Ye et al., 2011). In this research, we suspect that the peri-lesional nerve remodeling and outgrowth illustrated in Figures 3–5 were due to a similar inflammatory process, with the neurotrophic factors stimulating the neural tissues around the lesion.

To what extent and how the neural activity affect disease states and progression have been discussed in various tissues (Ondicova and Mravec, 2010). In rodent models, sympathectomy has been shown to decrease the size and invasiveness of tongue cancer by interfering the neurotransmitter and cytokine mediated interactions among the nerve, immune system, and tumor cells (Raju et al., 2007; Raju and Ibrahim, 2011). Also, in gastrointestinal cancer models, suppressing the adrenergic and cholinergic transmissions has been shown to ameliorate the course of tumor progression (Tatsuta et al., 1992; Schuller and Al-Wadei, 2010; Demir et al., 2012). In Figures 4, 5, we also demonstrate the intimate association between the chemically induced tongue lesion with the sympathetic and cholinergic nerves. Because of the direct nerve-lesion contacts, future studies on manipulation of the neural activities through neurotransmitters, including the neuropeptides (Kusakabe et al., 1998; Batbayar et al., 2004), and receptor antagonists will benefit our understanding of the disease mechanism in oral lesion/cancer progression for potential therapeutic intervention to supplement the current surgical treatment of oral cancer.

In summary, we developed a new tongue histological approach to reveal the 3-D features of the neuroanatomy. Prior to this research, the opaque tongue mucosa and muscles have hindered the observation of tongue innervation to study its role in oral lesion development. We prepared transparent tongue specimens by optical clearing for panoramic imaging and illustration of the tongue neural networks in health and disease. Future work will aim to study the human tongue innervation in the surgically removed tissues and oral biopsies.

### Conflict of interest statement

The authors declare that the research was conducted in the absence of any commercial or financial relationships that could be construed as a potential conflict of interest.

## Supplementary material

Figure S1 **Remodeling of pan-neuronal marker PGP9.5-labeled tongue innervation in response to injury (overlay of transmitted light and fluorescence image)**. The tile-scanning micrograph serves as a control image to confirm the PGP9.5 signals shown in Figure 3. This is demonstrated by the matched tongue microstructures and innervation patterns, such as the locations of the fungiform papillae and their innervation (green asterisks). The signals of the transmitted light micrograph were digitally reduced to enhance the presentation of the fluorescence signals.

Figure S2 **Tongue lesions and the peri-lesional nerves revealed by PGP9.5 staining. (A)** Papilloma with epithelial overgrowth. The skeletal muscle was intact at this stage of lesion formation. However, the immunostaining background was elevated at the muscle domain, which could be due to inflammation. **(B)** Peri-lesional innervation as lesions entering the muscle domain. At this stage, the lesions induced the remodeling of the surrounding nerves, creating a peri-lesional neural structure (asterisks). Zoom-in examination of the peri-lesional nerves is shown in Video S7.

Figure S3 **VAChT-labeled cholinergic nerves in normal and diseased tongues. (A)** Cholinergic innervation of mouse tongue. Two features are prominent: (1) the neuromuscular junctions (i.e., the VAChT^+^ foci) and (2) the coupling of VAChT^+ varicosities in space (the VAChT^+^ parasympathetic^ nerves). The in-depth projection reveals the coupling of varicosities, which otherwise cannot be easily portrayed by a standard 2-D micrograph. **(B–D)** Remodeling of tongue epithelium, muscle, and cholinergic innervation in response to injury. Panel **(B)** shows the transmitted light micrograph of the injured tongue. The cyan box in panel **(B)** is enlarged in panel **(C)** to examine the tongue lesion. The zoom-in examination shows the protrusion of epithelium created a lesion core (double-lined cyan box, enlarged in panel **D**) with condensed VAChT-labeled cholinergic neuromuscular junctions and parasympathetic nerve fibers (similar to the core shown in Figures 5D,E). Yellow lines in panel **(D)** indicate the lesion core boundaries.

Video S1 **In-depth visualization of tissue microstructure in the optically cleared mouse tongue.** Specimen was labeled with nuclear (green, propidium iodide) and membrane (red, DiD) dyes. By resolving the adjacent nuclei (an internal control in deep-tissue microscopy), we show that the photon penetration in the optically-cleared specimen was sufficient for subcellular-level microscopy. Imaging depth: 200 μm.

Video S2 **In-depth visualization and projection of tongue innervation at the mucosal boundary and muscle domain.** Specimen was labeled with nuclear dye(green) and pan-neuronal marker PGP9.5 (white). First part: gross view. Second part: zoom-in examination of muscle domain. The continuous display of the PGP9.5-labeled nerves and 360° projection of the image stack show the tongue innervation with a 3-D space continuum and without loss of signals at the center of the image stack (which otherwise would indicate limitation of antibody penetration in immunohistochemistry). Imaging depth: 200 μm.

Video S3 **In-depth visualization and 360° projection of vascular signals derived from the vessel-painted mouse tongue with optical clearing.** Imaging depth: 200 μm.

Video S4 **360-degree projection of the neurovascular complex at the core of filiform papilla.** Red: blood vessels. White: PGP9.5-labeled nerves.

Video S5 **Fly-through presentation of the microstructure and innervation of fungiform papilla.** Green: nuclei. White: PGP9.5-labeled nerves.

Video S6 **In-depth visualization and 360° projection of sensory nerves in taste bud.** Green: nuclei. Yellow: CGRP-labeled nerves. Imaging and projection depth: 105 μm.

Video S7 **Zoom-in examination of peri-lesional nerves shown in Figure S2B.** 360° projection was created to visualize the peri-lesional nerves from different angles.
