# Supplementary material for: 3-D neurohistology of transparent tongue in health and injury with optical clearing
Source: Front Neuroanat. 2013 Oct 22;7:36. doi: 10.3389/fnana.2013.00036 (PMC3805177; doi:10.3389/fnana.2013.00036)

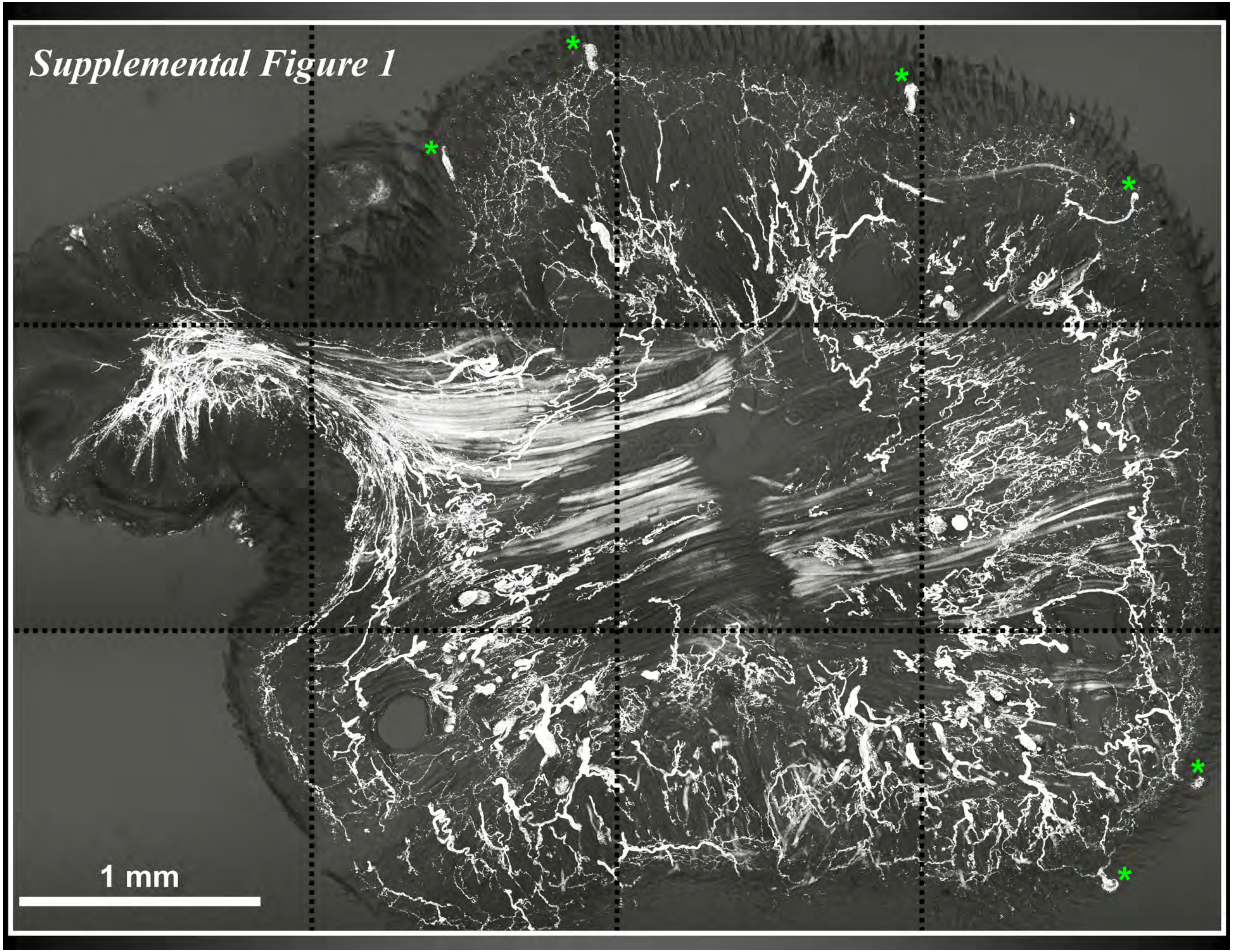
Supplemental Figure 1
*
*
*
*
*
*
1 mm

Supplemental Figure 2

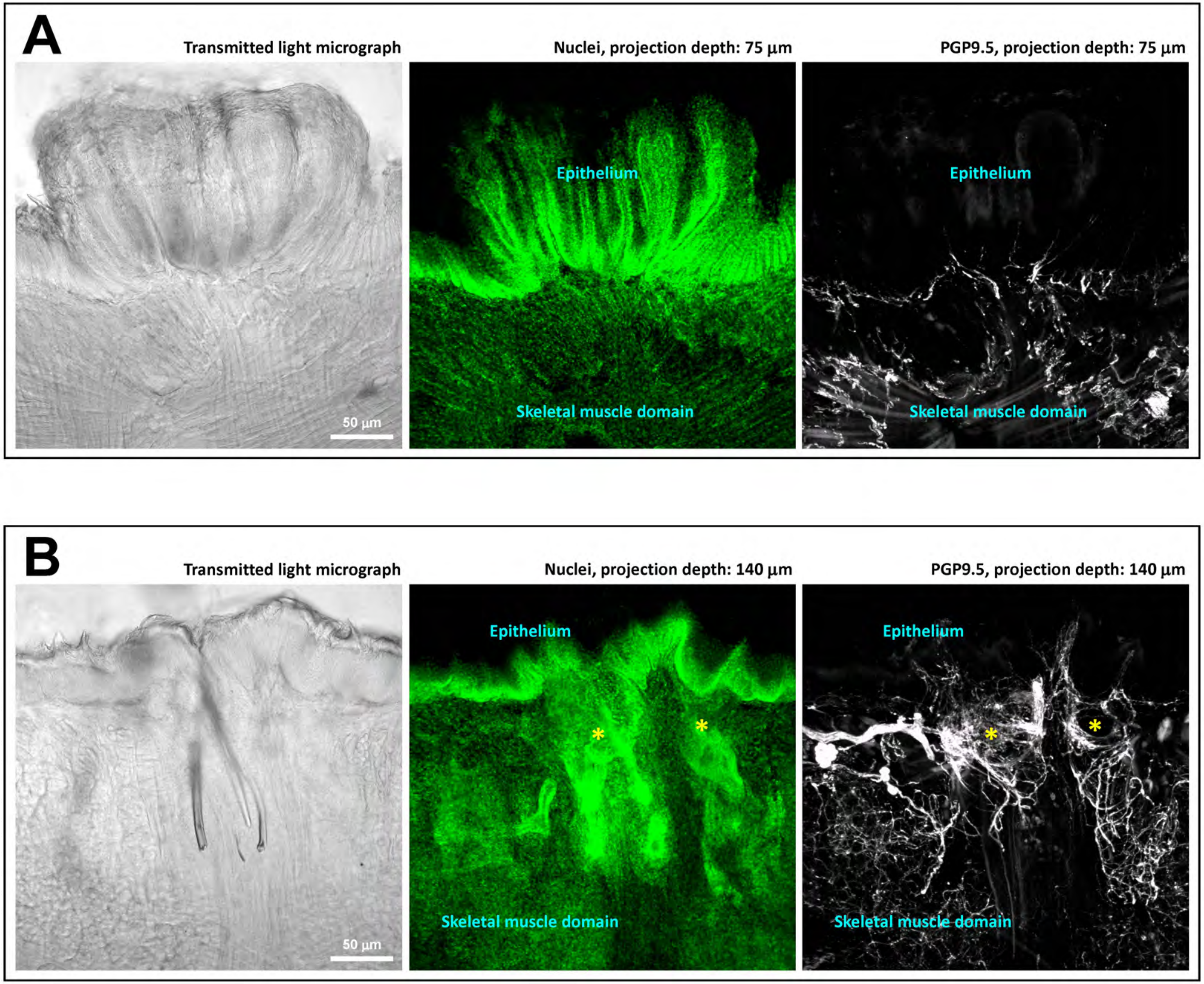

Supplemental Figure 3

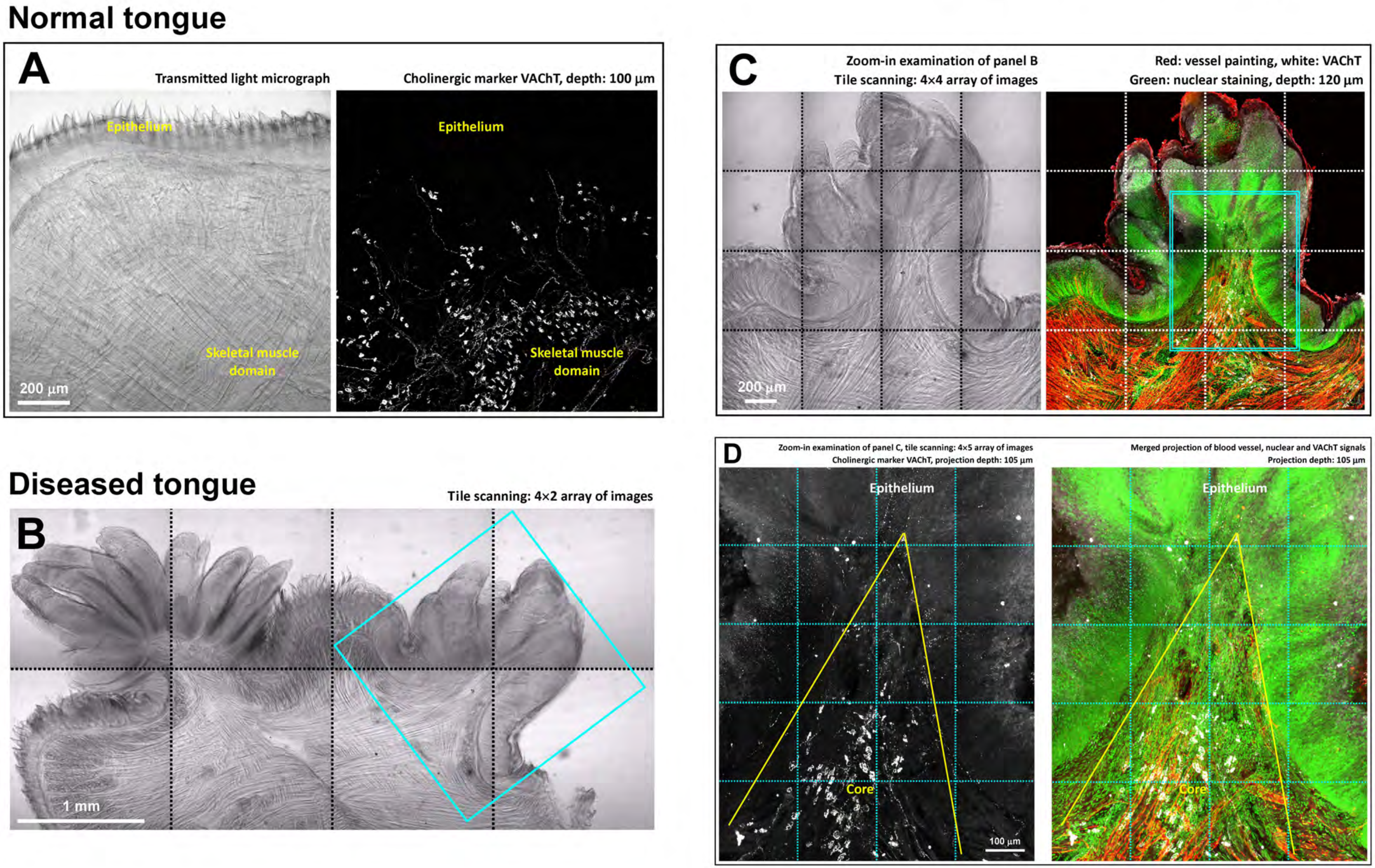

Supplement: Figure S1 — Remodeling of pan-neuronal marker PGP9.5-labeled tongue innervation in response to injury (overlay of transmitted light and fluorescence image). The tile-scanning micrograph serves as a control image to confirm the PGP9.5 signals shown in Figure 3. This is demonstrated by the matched tongue microstructures and innervation patterns, such as the locations of the fungiform papillae and their innervation (green asterisks). The signals of the transmitted light micrograph were digitally reduced to enhance the presentation of the fluorescence signals. [file Presentation1.PDF]
